# Inhibition of endoplasmic reticulum stress reverses synaptic plasticity deficits in striatum of DYT1 dystonia mice

**DOI:** 10.18632/aging.203413

**Published:** 2021-08-16

**Authors:** Huaying Cai, Linhui Ni, Xingyue Hu, Xianjun Ding

**Affiliations:** 1 Department of Neurology, Neuroscience Center, Sir Run Run Shaw Hospital, School of Medicine, Zhejiang University, Hangzhou 310016, China; 2 Department of Orthopaedic Surgery, Sir Run Run Shaw Hospital, School of Medicine, Zhejiang University, Hangzhou 310016, China

**Keywords:** DYT1 dystonia, endoplasmic reticulum stress, striatal synaptic plasticity, TUDAC

## Abstract

Background and objective: Striatal plasticity alterations caused by endoplasmic reticulum (ER) stress is supposed to be critically involved in the mechanism of DYT1 dystonia. In the current study, we expanded this research field by investigating the critical role of ER stress underlying synaptic plasticity impairment imposed by mutant heterozygous Tor1a^+/-^ in a DYT1 dystonia mouse model.

Methods: Heterozygous Tor1a^+/-^ mouse model for DYT1 dystonia was established. Wild-type (Tor1a^+/+^, N=10) and mutant (Tor1a^+/-^, N=10) mice from post-natal day P25 to P35 were randomly distributed to experimental and control groups. Patch-clamp and current-clamp recordings of SPNs were conducted with intracellular electrodes for electrophysiological analyses. Striatal changes of the direct and indirect pathways were investigated via immunofluorescence. Golgi-Cox staining was conducted to observe spine morphology of SPNs. To quantify postsynaptic signaling proteins in striatum, RNA-Seq, qRT-PCR and WB were performed in striatal tissues.

Results: Long-term depression (LTD) was failed to be induced, while long-term potentiation (LTP) was further strengthened in striatal spiny projection neurons (SPNs) from the Tor1a^+/-^ DYT1 dystonia mice. Spine morphology analyses revealed a significant increase of both number of mushroom type spines and spine width in Tor1a^+/-^ SPNs. In addition, increased AMPA receptor function and the reduction of NMDA/AMPA ratio in the postsynaptic of Tor1a^+/-^ SPNs was observed, along with increased ER stress protein levels in striatum of Tor1a^+/-^ DYT1 dystonia mice. Notably, ER stress inhibitors, tauroursodeoxycholic acid (TUDCA), could rescue LTD as well as AMPA currents.

Conclusion: The current study illustrated the role of ER stress in mediating structural and functional plasticity alterations in Tor1a^+/-^ SPNs. Inhibition of the ER stress by TUDCA is beneficial in reversing the deficits at the cellular and molecular levels. Remedy of dystonia associated neurological and motor functional impairment by ER stress inhibitors could be a recommendable therapeutic agent in clinical practice.

## INTRODUCTION

DYT1 dystonia, which is a complex neurological condition characterized by abnormal involuntary motors or postures [1], is usually attributed to a GAG base-pair deletion of *DYT1* (*Tor1a*) gene encoding Tor1a protein [2]. Although it has been recognized that the attack of movement disorder is usually between childhood and adolescence, what triggers the manifestation of clinical symptoms is still unknown [3]. Human studies have indicated synaptic plasticity impairments as major manifestations in dystonia patients [4]. Plasticity impairments, including functional and structural synaptic deficits, could lead to motor learning disabilities [5]. Furthermore, symptoms of abnormal motor learning have also been observed in Tor1a mutation (Tor1a^+/-^) carriers without evident clinical manifestation, which further supports the opinion that impaired synaptic plasticity might be an inherent feature of dystonia [6]. Notably, impaired striatal synaptic plasticity has been explored in dystonia rodent models, including heterozygous DYT1 knock-in mice [7, 8], which presented a remarkable similarity to the studies in dystonia patients [9]. Hence, the current evidence supports the judgement that DYT1 dystonia could be regarded as a neurodevelopmental disease with impaired striatal plasticity and motor dysfunction.

Although impairments in structural synaptic plasticity as well as functional counterpart have been revealed in rodent models of dystonia [10], similar to clinical demonstrations in dystonia patients, the potential mechanisms underlying synaptic plasticity impairments in dystonia are currently unclear. To date, several potential mechanisms have been put forward to be relevant, including abnormal reactive oxygen species (ROS) dynamics [11], neuroinflammation [12], and impaired brain-derived neurotrophic factor (BDNF) function [13]. However, the role of endoplasmic reticulum (ER) stress, which may result in critical cellular dysfunction and neuronal death [14], has been underestimated. Recently, researches have suggested that ER stress might play an important role in neurodegenerative and neurodevelopmental diseases [15, 16]. Two marker proteins of ER stress, including ATF-4 and CHOP, were found to be upregulated under condition of stress-related neuronal death [17]. Hence, we speculate that ER stress may contribute to the deficits in neuroplasticity and motor function in dystonia.

Whereas, currently, the research in the field of the association between ER stress and synaptic plasticity in dystonia is still lacking. Moreover, whether inhibition of ER stress could reverse functional and structural plasticity abnormalities in dystonia remains unclear. Here, this study confirmed impaired structural and functional plasticity in striatal synapses of Tor1a^+/-^ mice, which is paralleled by a significant increase in ER stress markers protein levels, along with the increased AMPA receptor function and the decreased NMDA/AMPA ratio in the postsynaptic of Tor1a^+/-^ spiny projection neurons (SPNs). Notably, ER stress inhibitors, TUDCA, could rescue LTD as well as AMPA currents. Our results illustrate potential mechanisms underlying synaptic plasticity impairments in Tor1a^+/-^ SPNs, demonstrating the close relationship between ER stress and plasticity abnormalities in dystonia, which could point out new directions for the treatment and prevention of dystonia.

## MATERIALS AND METHODS

### Heterozygous Tor1a^+/-^ mouse model for DYT1 dystonia

Animal experiments were performed in accordance with the guidelines for the use of animals in biomedical research [18]. The experimental procedures were approved by the Internal Institutional Review Committee of Zhejiang University (ZJU-2020-01-06). All efforts were made to reduce the number of animals and the degree of their suffering. Heterozygous Tor1a^+/-^ (N=10; Jackson Laboratory, ME, USA) and their wild-type C57BL/6J littermates (Tor1a^+/+^, N=10) were purchased. Age- and sex-matched wild-type and mutant mice from post-natal day P28 to P32 were randomly distributed to experimental and control groups. The potential influence of sex difference in neuromorphology and electrophysiological characteristics was excluded in preliminary experiments. Researchers conducting experiments and data analyses were unknown to the detailed information of mice genotype and treatment protocols.

### Brain slice preparation

The preparation of striatum slices from mice and genotyping was performed as previously described [19]. Briefly, mice were killed via cervical dislocation, striatum tissues were isolated from brains and were sliced with a vibratome in Krebs’ solution (126 mM NaCl, 2.5 mM KCl, 1.3 mM MgCl_2_, 1.2 mM NaH_2_PO_4_, 2.4 mM CaCl_2_, 10 mM glucose, 18 mM NaHCO_3_) saturated with 95% O_2_ and 5% CO_2_. Striatum slices in coronal and parasagittal patterns (200–300 mm) were incubated in Krebs’ solution at 27° C for a half hour. Next, striatum slices were shifted to the recording chambers for electrophysiological experiments.

### Patch-clamp recordings

Patch-clamp recordings was performed as previously described [20]. For the recordings of glutamatergic spontaneous excitatory postsynaptic current (sEPSC), SPNs were clamped in the presence of the GABA-A receptor antagonist PTX (50 mM) under HP=-70 mV. For the recordings of GABAergic spontaneous inhibitory postsynaptic current (sIPSC), under HP=+10 mV, SPNs were clamped in the presence of NMDAR antagonist MK801 (30 mM) and AMPAR antagonist CNQX (10 mM). In the presence of TTX (1 mM), both miniature excitatory postsynaptic current (mEPSC) and miniature inhibitory postsynaptic current (mIPSC) were recorded. In the presence of PTX (50 mM) under HP=-70 mV, paired-pulse ratio (PPR) was recorded by spurring two trains of stimuli at interstimulus interval (ISI) of 25-1000 ms. The NMDAR/AMPAR ratio was calculated to measure synaptic strength in the presence of PTX (50 mM) under HP=+40 mV. The EPSC component mediated by AMPAR was derived in the presence of MK-801 (30 mM), and then the EPSC component mediated by NMDAR was derived from the AMPAR component and the dual-component EPSC. The current and voltage (IV) relationships mediated by AMPAR and NMDAR were plotted in the presence of PTX (50 mM) combined with MK-801 (30 mM) or CNQX (10 mM). The rectification index (RI) was derived from the ratio of the mean amplitudes of EPSC at -70 mV or +40 mV.

### Current-clamp recordings

Current-clamp recordings of SPNs were performed as previously described [21]. Excitatory postsynaptic potentials (EPSPs) were recorded in the presence of PTX (50 mM). Three trains of high frequency stimulation (HFS, 100 Hz) were spurred at to induce long-term depression (LTD). Magnesium (Mg^2+^) was filtered to induce long-term potentiation (LTP). Average amplitude of EPSP was presented as percentage of average amplitude of EPSP in control group before HFS delivery.

### Western blotting, quantitative real-time PCR and RNA-Seq analysis

To quantify postsynaptic signaling proteins in striatum, tissue homogenate of striatum was prepared for western blotting, quantitative real-time PCR and RNA-Seq analysis as previously described [22]. Detailed information was presented in Supplemental Materials.

### Immunofluorescence

Striatal changes of the direct and indirect pathways were investigated via immunofluorescence (IF) as previously described [23]. In brief, striatum slices (about 20~30 μm) were dehydrated with gradient alcohol. the following primary antibodies were applied: rabbit monoclonal anti-DARPP32 (1:100, ab40801, Abcam, MA, USA), rabbit polyclonal anti-Enkephalin (5 μg/ml, ab85798, Abcam). Images were obtained with a confocal laser scanning microscope (LSM700, Carl Zeiss, Germany) and analyzed via ImageJ software.

### Spine morphology

Golgi-Cox staining was conducted to observe spine morphology of SPNs, as previously described [24]. Briefly, brain tissues were stained via the Rapid Golgi Staining Kit (FD Neuro-Technologies). Then, SPNs from the striatum were observed under Zeiss Microscope. Apical dendrites with spines from 10 neurons for each slice were traced through a 60× lens to measure spine density. Next, the number of spines per 10 μm length was calculated. Furthermore, spine subtypes were defined via the relative proportion of the length, head diameter, and neck diameter of spine.

### Drug treatment

Tauroursodeoxycholic acid (TUDCA, Sigma-Aldrich, MO, USA) was dissolved in phosphate-buffered saline (PBS) of pH 7.4 at a concentration of 7.8 mg/ml according to the manufacturer protocol. As previously described [25], TUDCA was daily intraperitoneally injected (100 mg/kg) for 14 days. The mice were randomly assigned into four groups: the PBS-treated Tor1a^+/+^ mice (N=5), TUDCA-treated Tor1a^+/+^ mice (N=5), PBS-treated Tor1a^+/-^ mice (N=5), and TUDCA-treated Tor1a^+/-^ mice (N=5). Data for PBS-treated mice was not shown in the results.

### Statistical analysis

Data were analyzed with ClampFit (Molecular Devices, CA, USA) and GraphPad Prism (GraphPad Software, CA, USA) software. All data were obtained from at least three independent experiments and are represented as mean ± SEM. Statistical significance was evaluated using Student’s t test, one-way ANOVA or two-way ANOVA with post-hoc test for group comparisons. The statistical significance was set at *P*<0.05.

### Availability of data and material

Data and material are available on request.

### Compliance with ethical standards

Animal experiments were performed in accordance with the guidelines for the use of animals in biomedical research. The experimental procedures were approved by the Internal Institutional Review Committee of Zhejiang University (ZJU-2020-01-06). All efforts were made to minimize the number of animals and their suffering.

## RESULTS

### Altered striatal long-term synaptic plasticity in Tor1a^+/-^ mice

**Figure 1:**
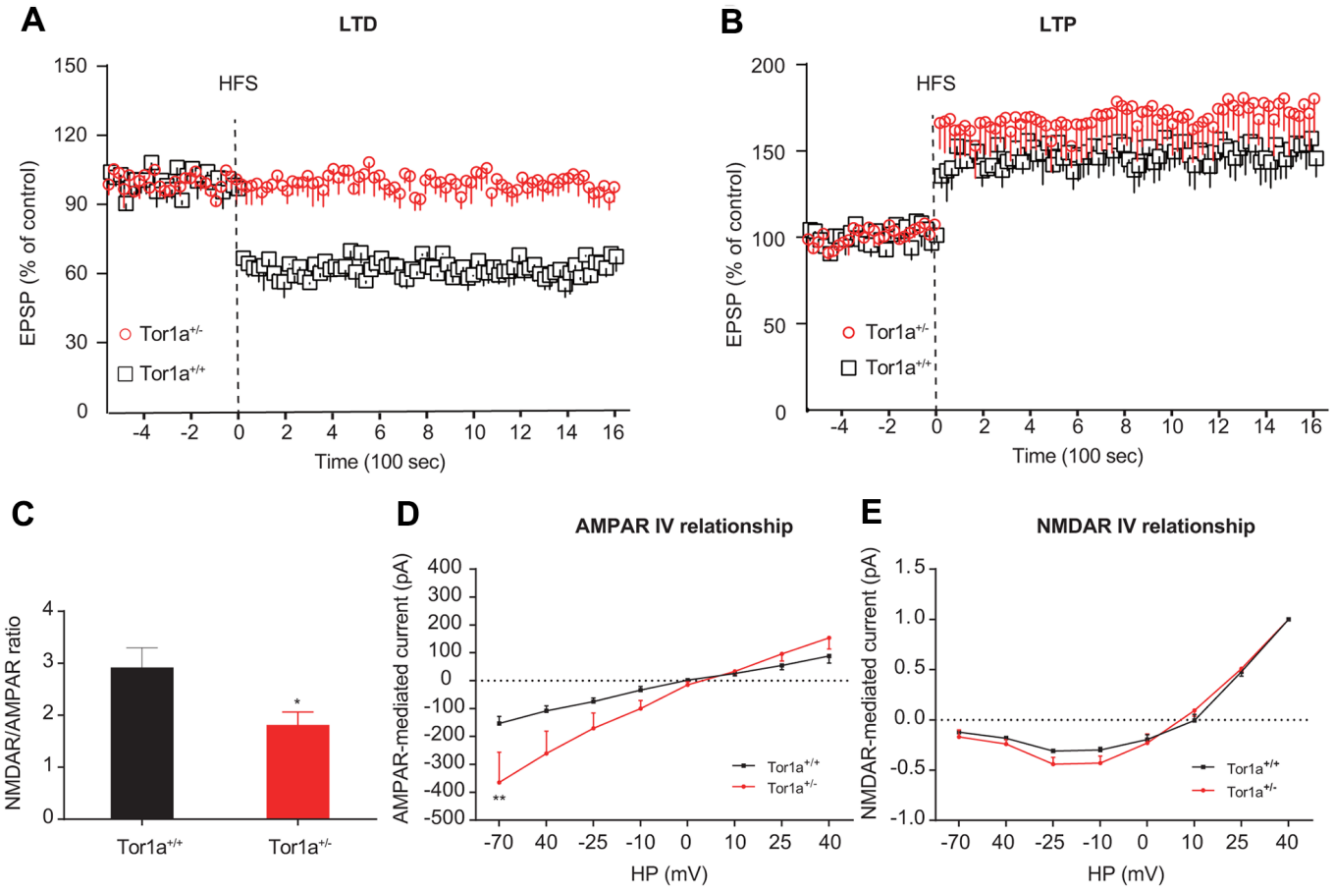
**Altered striatal long-term synaptic plasticity in Tor1a^+/-^ mice.** (**A**) Time-course of striatal LTD expression in SPNs from Tor1a^+/+^ and Tor1a^+/-^ mice. HFS protocol induced LTD in SPNs recorded from Tor1a^+/+^ mice (*P*<0.05), but not from Tor1a^+/-^ mice (*P*>0.05). (**B**) Time-course of striatal LTP expression in SPNs from Tor1a^+/+^ and Tor1a^+/-^ mice. In Tor1a^+/-^ SPNs LTP showed a tendency to increase compared with wild-type SPNs. (**C**) Summary plot of NMDA/AMPA current ratio calculated in SPNs from Tor1a^+/+^ and Tor1a^+/-^ mice. A significant decrease of NMDA/AMPA ratio was detected in Tor1a^+/-^ mice, compared to Tor1a^+/+^ mice. (**D**) AMPAR-mediated currents recorded at different HP in Tor1a^+/+^ and Tor1a^+/-^ SPNs. The I-V relationship showed a significant increase in the current recorded at more hyperpolarized range from Tor1a^+/-^ SPNs (HP= -70 mV, *P*<0.01). (**E**) Normalized IV relationships of NMDAR-mediated currents showed no difference between genotypes (*P* >0.05). In each group, five mice were used (N=5), and three independent electrophysiological recordings were conducted for each mouse (n=3). *P*<0.05 was considered to be statistically significant.

We firstly explored the basic electrophysiological characteristics of SPNs from Tor1a^+/+^ and Tor1a^+/-^ mice. SPNs from both mice did not exhibit significant discrepancy in the basic electrophysiological characteristics (Supplementary Figure 1). Then, we explored the characteristics of LTD and LTP from Tor1a^+/+^ and Tor1a^+/-^ mice. In Tor1a^+/-^ SPNs, the HFS stimulation could not induce a synaptic depression (Figure 1A; *P*>0.05), while a robust LTD was elicited in Tor1a^+/+^ SPNs (*P*<0.05). Furthermore, the LTP induction protocol induced a stable LTP in Tor1a^+/+^ SPNs (Figure 1B; *P*<0.05). Notably, an increasing trend was found in Tor1a^+/-^ SPNs LTP, compared with wild-type SPNs, indicating a hyperexcitability (Tor1a^+/+^: 148.99 ± 12.17 of pre-HFS; Tor1a^+/-^: 171.82 ± 18.29 of pre-HFS; *P*<0.05).

At excitatory synapses, NMDARs form a unique cluster mainly at the center of the postsynaptic density (PSD), while AMPARs segregate in clusters surrounding the NMDARs, and the balance of them affects the synaptic transmission properties of a unitary synapse [26]. Hence, the NMDAR/AMPAR current ratio in SPNs were recorded, in order to unmask the electrophysiological physiology of AMPARs and NMDARs currents in SPNs from both Tor1a^+/+^ and Tor1a^+/-^ mice. Significant decrease of the NMDAR/AMPAR current ratio was revealed in Tor1a^+/-^ SPNs, compared to that of Tor1a^+/+^ SPNs (Figure 1C; *P*<0.05), indicating an enhanced AMPARs abundance relative to NMDARs quantity. Under different HP, both AMPAR-mediated and NMDAR-mediated currents were recorded in Tor1a^+/+^ and Tor1a^+/-^ SPNs. Under hyperpolarized voltage ranges (-70~40 mV), increased AMPAR-mediated current was observed in Tor1a^+/-^ SPNs, compared to Tor1a^+/+^ SPNs (Figure 1D). However, no significant difference was revealed in NMDAR-mediated current (Figure 1E).

### Altered molecular markers and morphology of synapse in Tor1a^+/-^ mice

**Figure 2:**
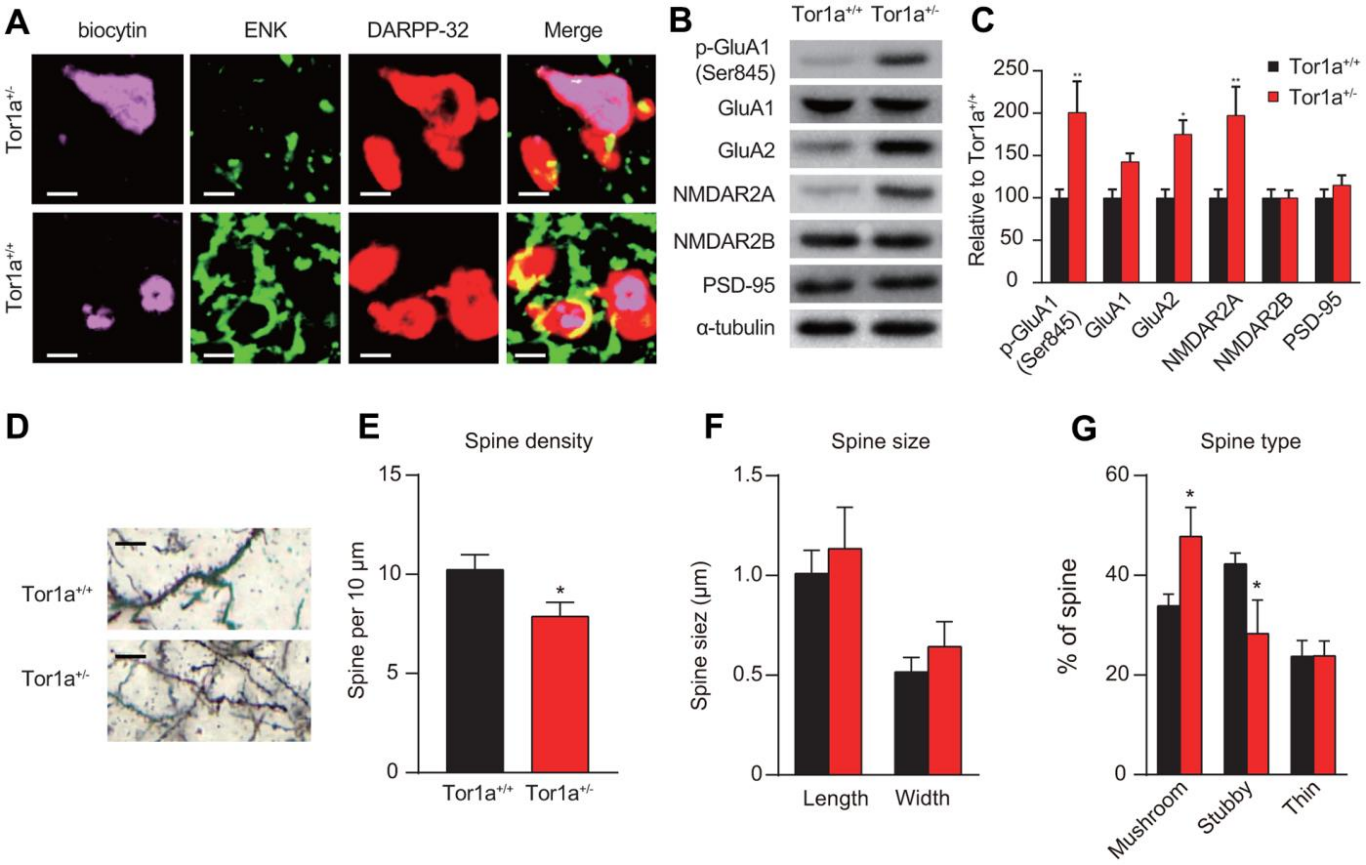
**Altered molecular markers and morphology of synapse in Tor1a^+/-^ mice.** (**A**) Representative confocal images from two SPNs recorded in Tor1a^+/-^ and Tor1a^+/+^ slices (scale bar: 10 μm). Recording electrodes were filled with biocytin (pink) and SPNs were immunolabelled with anti-ENK (green) and anti-DARPP-32 (red). ENK-negative SPNs from Tor1a^+/-^ slices failed to induce LTD. (**B**) WB analysis for p-GluA1 (Ser845), GluA1, GluA2, NMDAR2A, NMDAR2B, PSD-95 and α-tubulin in Tor1a^+/-^ and age-matched Tor1a^+/+^ mice. (**C**) Histogram showed the quantification of protein levels following normalization on α-tubulin in Tor1a^+/-^ and age-matched Tor1a^+/+^ mice. All values are mean ± SEM expressed as % of Tor1a^+/+^ mice. (**D**) Representative images showed spine morphology of Tor1a^+/-^ and age-matched Tor1a^+/+^ mice (scale bar: 10 μm). (**E**) Histogram represented dendritic spine density in Tor1a^+/-^ and Tor1a^+/+^ SPNs. Tor1a^+/-^ SPNs exhibited an overall decrease of dendritic spine density (*P*<0.05). (**F**, **G**) Histograms showed the quantification of dendritic spine size (**F**, spine length and head width) and dendritic spine type (**G**, mushroom, stubby, thin) in Tor1a^+/-^ and age-matched Tor1a^+/+^ mice. A larger number of mushroom-type spines and a concomitant smaller number of stubby-type spines were found in Tor1a^+/-^ SPNs (both *P*<0.05). In each group, five mice were used (N=5), and three independent experiments were conducted for each mouse (n=3). *P*<0.05 was considered to be statistically significant.

To identify potential mechanism of direct- and indirect-pathway in SPNs, confocal images were obtained from two SPNs recorded in Tor1a^+/-^ and Tor1a^+/+^ slices. Recording electrodes were filled with biocytin (pink) and SPNs were immunolabelled with anti-ENK (green) and anti-DARPP-32 (red). Immunofluorescence staining indicated that ENK-negative SPNs from Tor1a^+/-^ failed to induce LTD (upper panel of Figure 2A), while SPNs from Tor1a^+/+^ slices exhibited to be ENK-positive (lower panel of Figure 2A), indicating a possible inhibition of indirect-pathway in SPNs from Tor1a^+/-^ slices.

Moreover, the expressions of AMPAR and NMDAR subunits were evaluated using WB analysis. The levels of AMPAR subunits (p-GluA1-Ser845 and GluA2) were significantly increased, but not for GluA1, in the striatal tissues of Tor1a^+/-^ mice, compared to that of wild-types (Figure 2B, 2C; both *P*<0.05). Interestingly, an increase of NMDAR2A but not NMDAR2B subunit was observed in the striatal tissues of Tor1a^+/-^ mice (*P*<0.05). However, we didn’t observe a significant alteration in PSD-95 (*P*>0.05), which is known to be involved in maturation of excitatory synapses [27].

In addition, we further evaluated spine morphology in Tor1a^+/-^ and Tor1a^+/+^ SPNs. Dendritic spine density was decreased in Tor1a^+/-^ SPNs (Figure 2D, 2E). In detail, the number of mushroom-type spines was significantly increased (Figure 2G; *P*<0.05), while the number of stubby-type spines was decreased (Figure 2G; *P*<0.05). However, no significant difference was found for dendritic spine length and head width (Figure 2F; *P>*0.05). Consequently, these alterations were associated to early spine maturation.

### Abnormal eIF2α signaling and ER stress in Tor1a^+/-^ mice

**Figure 3:**
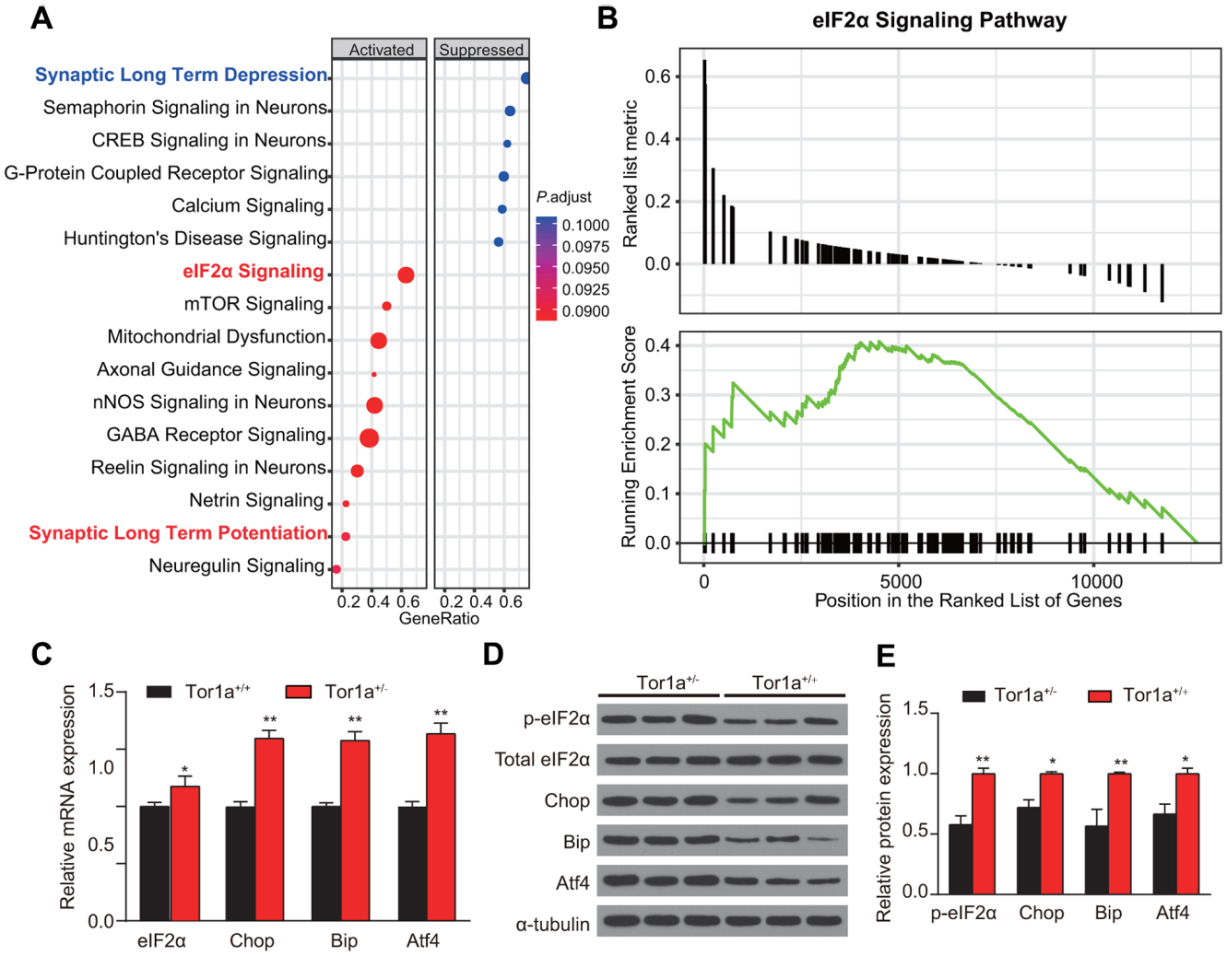
**Abnormal eIF2α signaling and ER stress in Tor1a^+/-^ mice.** (**A**) Ingenuity pathway analysis (IPA) was completed to identify significantly dysregulated canonical pathways. The top 15 pathways generated with the DEGs. (**B**) Gene Set Enrichment Analysis (GSEA) was applied to further confirm the up-regulated eIF2α signaling in Tor1a^+/-^ mice. (**C**) Levels of mRNA in striatal lysates were measured by RT-qPCR. (**D**, **E**) Representative western blots of striatal lysates (**D**). Quantification of protein expression in striatum as shown (n: 3 per group). Data are represented as mean ±SEM. In each group, five mice were used (N=5), and three independent experiments were conducted for each mouse (n=3). *P*<0.05 was considered to be statistically significant.

Impairment of eIF2α signaling, which has been regarded to be related with cellular stress responses and synaptic plasticity, was identified in patients with idiopathic dystonia [8]. Hence, we speculated that mutant Tor1a in DYT1 mice might lead to basal abnormalities in the eIF2a signaling and ER stress. We tried to validate this hypothesis in heterozygous Tor1a^+/-^ mouse model for DYT1 dystonia. After RNA-Seq analysis of striatal tissue was performed, the DEGs were then analyzed using Ingenuity Pathway Analysis (IPA) to identify dysregulated canonical pathways (Figure 3A). As expected, eIF2a signaling was one of the most up-regulated pathways in Tor1a^+/-^ mouse, which was further confirmed by GSEA analysis (Figure 3B). Notably, synaptic plasticity associated pathways were also dysregulated, with down-regulated LTD and up-regulated LTP.

Tor1a expression has been suggested to alter the cellular response to acute ER stress [28]. Whereas, it has not been validated in striatum of DYT1 knock-in mice. RT-qPCR revealed a significant increase in levels for eIF2α, Chop, Bip, and Atf4 mRNA in Tor1a^+/-^ mice compared with the wild-type controls (Figure 3C, all *P*<0.05), indicating induction of ER stress. Similarly, WB for expression of the components of eIF2α signaling further confirmed the activation of eIF2α signaling and induction of ER stress (Figure 3D, 3E).

### ER stress inhibitor reversed long-term memory deficit in Tor1a^+/-^ mice

**Figure 4:**
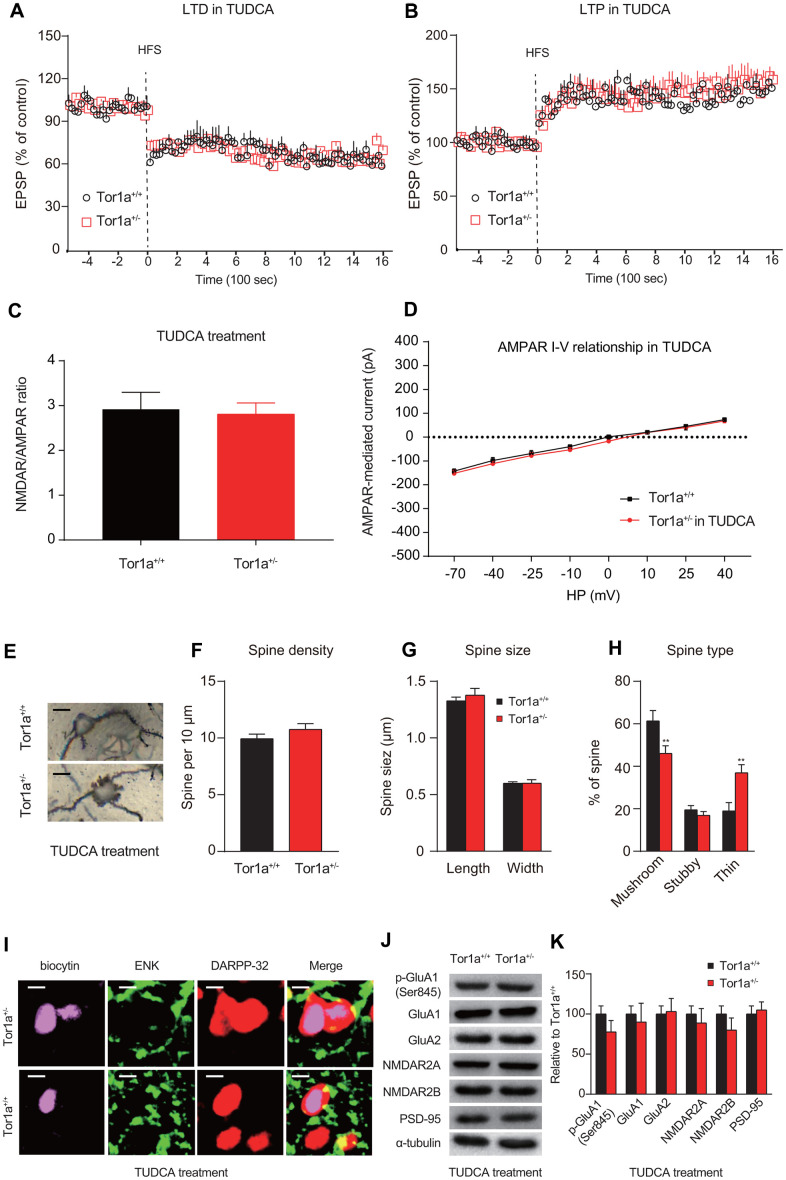
**ER stress inhibitor rescues long-term memory deficit in Tor1a^+/-^ mice.** (**A**) Time-course of striatal LTD expression in SPNs from Tor1a^+/+^ and Tor1a^+/-^ mice. After *in vivo* treatment with TUDCA, the HFS protocol induced striatal LTD expression in Tor1a^+/-^ mice. (**B**) Time-course of striatal LTP expression in SPNs from Tor1a^+/+^ and Tor1a^+/-^ mice. LTP amplitude in Tor1a^+/-^ mice was comparable to that of SPNs from Tor1a^+/+^ littermates. (**C**) Summary plot of NMDA/AMPA current ratio calculated in SPNs from Tor1a^+/+^ and Tor1a^+/-^ mice. treatment with TUDCA normalized the NMDAR/AMPAR ratio in Tor1a^+/-^ mice (*P*>0.05). (**D**) AMPAR-mediated currents recorded at different HP in Tor1a^+/+^ and Tor1a^+/-^ SPNs. The IV curve of AMPAR-EPSC also revealed no significant difference between genotypes (*P*>0.05). (**E**) Representative images showed spine morphology of Tor1a^+/-^ and Tor1a^+/+^ SPNs. (**F**–**H**) Histogram representing the quantification of dendritic spine density (**F**), dendritic spine size (**G**, spine length and head width) and dendritic spine type (**H**, mushroom, stubby, thin) in Tor1a^+/-^ and Tor1a^+/+^ SPNs. (**I**) Representative confocal images from two SPNs recorded in Tor1a^+/-^ and Tor1a^+/+^ slices after treatment of TUDCA (scale bar: 10 μm). Recording electrodes were filled with biocytin (pink) and SPNs were immunolabelled with anti-ENK (green) and anti-DARPP-32 (red). (**J**) WB analysis for p-GluA1 (Ser845), GluA1, GluA2, NMDAR2A, NMDAR2B, PSD-95 and α-tubulin in striatum tissues of Tor1a^+/-^ and age-matched Tor1a^+/+^ mice after treatment of TUDCA. (**K**) Histogram shows the quantification of protein levels following normalization on α-tubulin in Tor1a^+/-^ and age-matched Tor1a^+/+^ mice. In each group, five mice were used (N=5), and three independent electrophysiological recordings or experiments were conducted for each mouse (n=3). *P*<0.05 was considered to be statistically significant.

To investigate whether activation of ER stress was involved in the long-term synaptic plasticity deficits in Tor1a^+/-^ mice, eIF2α signaling was selectively blocked by the TUDCA. Firstly, we investigated the effect of TUDCA on the levels of ER stress markers. As expected, the results of RT-qPCR and WB consistently revealed that the levels of these proteins (p-eIF2α, Chop, Bip, and Atf4) in striatal tissues of Tor1a^+/-^ mice were significantly reduced after treatment with TUDCA (Supplementary Figure 2). After repetitive treatment with TUDCA (100 mg/kg, intraperitoneally), corticostriatal LTD was completely rescued in Tor1a^+/-^ mice (Figure 4A; *P*<0.05). In addition, after TUDCA treatment, the amplitude of LTP was reduced in Tor1a^+/-^ mice (Figure 4B; *P*>0.05). Similarly, treatment with TUDCA completely normalized the NMDAR/AMPAR ratio in Tor1a^+/-^ mice (Figure 4C; *P*>0.05). The IV curve of AMPAR-EPSC also revealed no significant difference between genotypes (Figure 4D; *P*>0.05).

Furthermore, we also evaluated spine morphology in Tor1a^+/+^ and Tor1a^+/-^ SPNs after treatment with TUDCA (Figure 4E). Tor1a^+/-^ SPNs returned to a normalization of spine density (Figure 4G; *P*>0.05) and of spine size (Figure 4F; *P*>0.05), compared to Tor1a^+/+^ SPNs. Interestingly, the number of mushroom spines in Tor1a^+/-^ SPNs sharply decreased to a lower level than that of Tor1a^+/+^ SPNs, along with a significant increase of thin spines (Figure 4H; *P*<0.05), which could be due to an over-reaction to TUDCA in Tor1a^+/-^ SPNs. Further evidence also supported that TUDCA could reverse alteration molecular markers for synapse plasticity. In a manner like SPNs from Tor1a^+/+^ slices, SPNs from Tor1a+/- slices also revealed to be ENK-positive (Figure 4I), indicating a release of inhibition of indirect-pathway in SPNs from Tor1a^+/-^ slices after TUDCA treatment. As WB analyses showed, the levels of AMPAR (p-GluA1-Ser845, GluA1, and GluA2) and NMDAR (NMDAR2A and NMDAR2B) subunits, as well as PSD95, were normalized in the striatal tissues of Tor1a^+/-^ mice after TUDCA treatment (Figure 4J, 4K). In addition, the intracellular Ca^2+^ release induced by Tor1a^+/-^ was markedly alleviated by the ER stress inhibitor TUDCA (Supplementary Figure 3). These data suggest that activated ER stress is a potential cause of the dysfunction of AMPARs in SPNs, further resulting in synaptic plasticity deficits in Tor1a^+/-^ mice.

## DISCUSSION

Although the role of activated ER stress in DYT1 dystonia has been previously explored *in vitro* and *in vivo* [29–31], these studies revealed to be limited for the study of ER stress via DYT1 dystonia mouse model. In the current study, we expanded this research field by investigating the role of ER stress underlying synaptic plasticity impairment in mutant heterozygous Tor1a^+/-^ in a DYT1 dystonia mouse model. We obtained evidence from different aspects to support this conclusion. First, Tor1a^+/-^ mice revealed activated ER stress in the striatum accompanied by upregulation of p-eIF2α, Chop, Bip, and Atf4, sensitive indicators of ER stress [32, 33]. The ER stress inhibitor, TUDCA, could specifically reverse these effects in Tor1a^+/-^ mice. Second, structural impairments in the synapses in SPNs from Tor1a^+/-^ mice were also repaired by inhibition of ER stress. Third, at the electrophysiology level, inhibition of ER stress by TUDCA could completely rescue the impairment in LTD in Tor1a^+/-^ mice. Taken together, the above results indicated that synaptic plasticity impairment in Tor1a^+/-^ mice could be partially due to ER stress in the striatum, which further leads to deficits of structural and functional synaptic plasticity. Inhibition of activated ER stress might correct these alterations and is therefore beneficial for motor function recovery in DYT1 dystonia. This study further extends our understanding of molecular mechanisms underlying dystonia, and establishes a new functional paradigm to evaluate the inhibitor of ER stress to compensate for mutant Tor1a, which may be beneficial for neuronal function.

Recent researches have proposed a correlation between eIF2α signaling and dystonia in DYT1 rodent model and dystonia patients [8, 34]. In this study, we conducted a comprehensive transcriptomic analysis in the striatal tissues from DYT1 dystonia mice brain, which provided another piece of bioinformatic evidence supporting this association. Whereas, it remains unknown whether dysregulation of eIF2α signaling plays a pathogenic role in DYT1 dystonia, or it is unrelated to DYT1 dystonia simply as a byproduct of Tor1a^+/-^ mutant. Intriguingly, the electrophysiological deficits and synapse morphology alterations observed in Tor1a^+/-^ mice, are partially or completely rescued after inhibition of the eIF2a signaling by TUDCA, indicating a potential pathogenic relevance. In addition, RT-PCR and WB analyses also found transcriptional dysregulation of eIF2α signaling in striatum tissues from Tor1a^+/-^ mice. Hence, all these findings confirmed a relationship between DYT1 dystonia and eIF2α dysregulation, which might further lead to an abnormal response to ER stress in Tor1a^+/-^ mice. On the one hand, the role of eIF2α signaling has been dependent on homeostatic balance to affect neurobiological processes, including synaptic plasticity and neurite maturation [35, 36]. Since DYT1 dystonia is a type of neurodevelopmental disorders, Tor1a^+/-^ mutation might lead to the pathogenic process through ER stress-dependent way. On the other hand, eIF2α dysregulation may contribute to DYT1 dystonia [37]. However, all these suspects are critical questions waiting to be addressed in the future, as they harbor significant potential in illuminating the mechanism of DYT1 dystonia pathology.

Although one of the most remarkable detrimental results of ER stress in striatum is neuronal apoptosis [38], we further observed subtle alterations in the synapse morphology induced by ER stress, such as reduction in the spine density and increase in the number of mushroom-type spines. Consistently, a previous study also suggested that ER stress disturbance could lead to synaptic impairments and interrupt neurotransmission related to synaptic morphology [39]. The finding that the density of spines is significantly reduced in Tor1a^+/-^ SPNs further supports the notion that subtle functional and structural alterations in SPNs play important roles in neurological dysfunction in dystonia. Notably, our finding of no significant alteration in PSD-95 expression, which was considered to be associated with synapse maturation [40], could be controversial. This may be associated with chaotic unfolded proteins reactions corresponding to ER stress. Besides, ubiquitin-proteasome system activated by ER stress could induce protein degradation and might play a role in maintaining the homeostasis of postsynaptic proteins, such as PSD-95 [41].

As described in the current study, along with molecular and structural changes at striatal synapses, electrophysiological alterations also revealed in SPNs from Tor1a^+/-^ mice. Striatal LTP is suspected to be dependent on the activation of NMDAR, while LTD depends on AMPAR [42, 43]. The electrophysiological and WB analyses indicated a significant increase in AMPAR-mediated currents, along with the decreased NMDAR/AMPAR ratio in SPNs from Tor1a^+/-^ mice. A potential regulatory mechanism for synaptic plasticity relies on the balance between synaptic insertion into the postsynaptic membrane and glutamate receptors removal from the counterparts [44]. In brief, dysregulated AMPA receptor in the postsynaptic membrane could lead to the dyshomeostasis of excitatory synapses. As our results presented, AMPAR subunits (GluA1 and GluA2) in the post-synaptic membrane of Tor1a^+/-^ SPNs significantly increased, indicating an increased AMPA receptor abundance. Notably, the phosphorylation of GluA1-Ser845 (p-GluA1) is over-expressed in Tor1a^+/-^ mice, which is consistent with a well-established relationship between p-GluA1 and LTP [42]. In detail, p-GluA1 mainly takes part in LTP, which is associated with GluA1-containing AMPARs, and might play an indispensable role in post-synaptic membrane stabilization for AMPARs. Hence, we have reasons to believe that the dysregulation of striatal AMPARs may be involved in the loss of LTD in Tor1a^+/-^ mice.

Alterations of spine morphology are supposed to be dependent on the composition of NMDARs and AMPARs as well as synaptic plasticity function, which further determine the long-term structural plasticity [45]. In primary hippocampal neurons, an increase in the number of mushroom spines was found to be accompanied by a decrease in spine density, which is a sign of ‘hyperexcitability’. It has been acknowledged that the NMDAR2 subunits of NMDARs become prevalently existed and abundant throughout the striatum after more than two postnatal weeks [46, 47]. In addition, a previous research has proposed that there is a positive correlation between spine size and AMPAR-mediated current at synaptic post-membranes [48]. In consistent with these findings, in Tor1a^+/-^ mice, we also found the levels GluA1 and GluA2 subunits of AMPARs increased accompanied by an increase in spine head width and the number of mushroom spines.

The current study firstly conducted a comprehensive investigation on whether ER stress contributes to underlying impairment in neuroplasticity base on a DYT1 dystonia mouse model. The results demonstrated that ER stress was induced by upregulation of Atf-4 and Chop. In addition, spine morphology of striatal synapses was change probably due to ER stress-induced protein degradation, which resulted in the impairment of synaptic plasticity. These effects contribute to the loss of LTD and motor deficits. Notably, TUDCA, an inhibitor of ER stress, could rescued LTD and AMPAR currents alteration. Our study illuminates that ER stress inhibitor could be a potential treatment means for neuronal protection in dystonia.

In conclusion, the current study suggests that ER stress might play a key role in synapse plasticity deficits in Tor1a^+/-^ striatum SPNs. Accumulation of misfolded proteins (eIF2α, Chop, Bip, and Atf4) and altered levels of AMPAR and NMDAR subunits could result in disordered neurological functions and impaired synaptic plasticity in striatum that underlie motor function and coordination. Inhibition of the ER stress by TUDCA is beneficial in reversing the deficits at the cellular and molecular levels. Considering the everlasting efforts in pharmaceutical industries and researchers to find out more therapeutic treatments in ameliorating dystonia, remedy of dystonia associated neurological and motor functional impairment by ER stress inhibitor could be a recommendable therapeutic agent in clinical practice.

## Supplementary Material

Supplementary Figures
