# Supplementary material for: Inhibition of endoplasmic reticulum stress reverses synaptic plasticity deficits in striatum of DYT1 dystonia mice
Source: Aging (Albany NY). 2021 Aug 16;13(16):20319–34. doi: 10.18632/aging.203413 (PMC8436893; doi:10.18632/aging.203413)
Supplement: Supplementary Figures [file aging-13-203413-s001.pdf]

SUPPLEMENTARY FIGURES

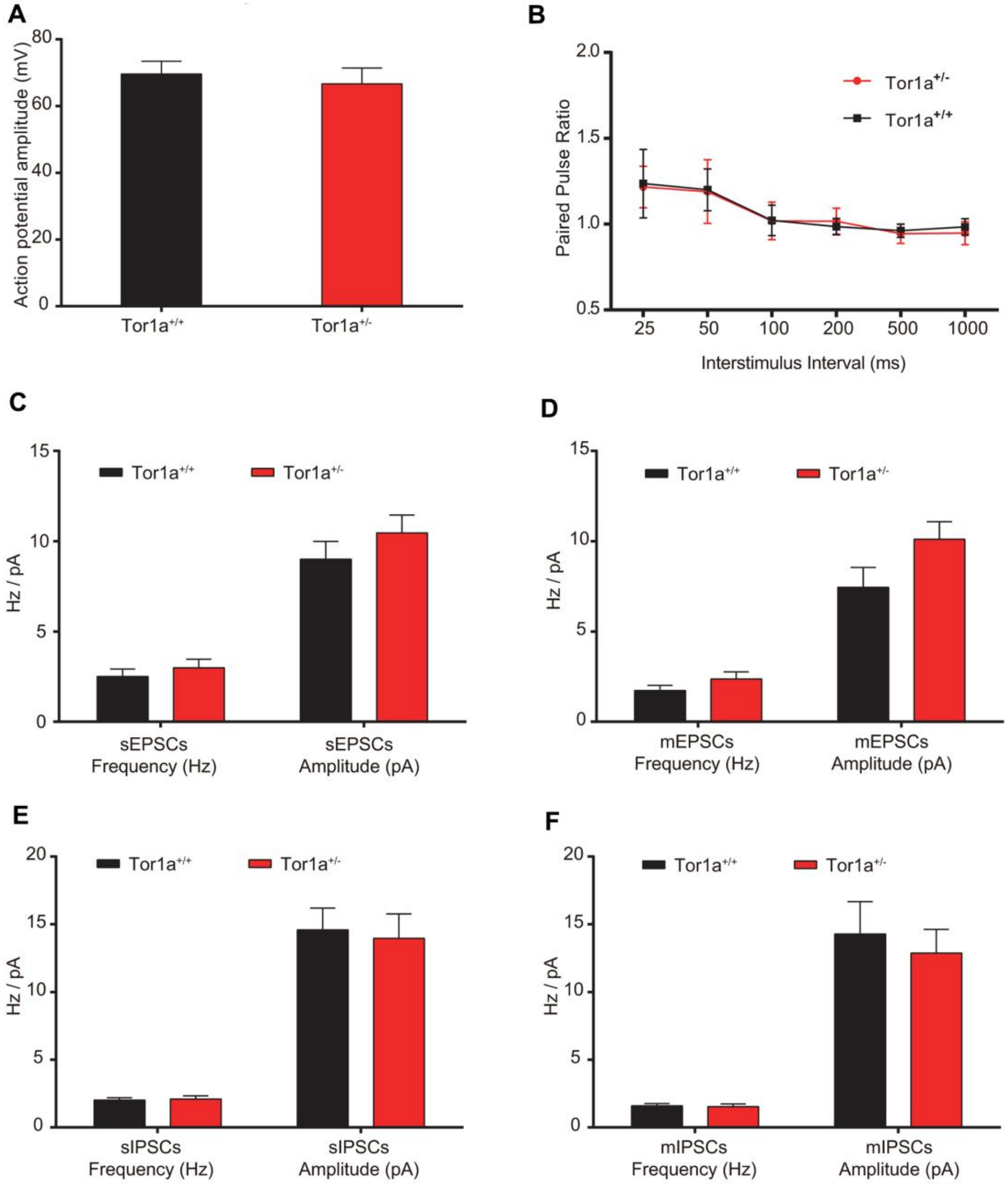

Supplementary Figure 1. Intrinsic and synaptic properties of SPNs from juvenile mice. (**A**) Depolarizing (+600 pA) and hyperpolarizing (200 pA) current steps caused tonic action potential discharge in SPNs recorded from Tor1a$^{+/+}$ (black) and Tor1a$^{+/-}$ (red) mice. (**B**) Paired-pulse ratio (PPR) showed similar facilitation in both genotypes. Short ISI (25-50 ms) of paired synaptic stimulation could induce PPF in both genotypes ($P<0.05$), whereas longer ISI (100-1000 ms) failed ($P>0.05$). (**C**) Glutamatergic sEPSCs recordings in PTX from SPNs of Tor1a$^{+/+}$ and Tor1a$^{+/-}$ mice showed no significant difference between genotypes in frequency and amplitude (both $P>0.05$). (**D**) Glutamatergic mEPSCs recordings in PTX plus TTX from SPNs of Tor1a$^{+/+}$ and Tor1a$^{+/-}$ mice showed no significant difference between genotypes in frequency and amplitude (both $P>0.05$). (**E**) GABAergic sIPSCs recordings in MK-801 and CNQX from SPNs of Tor1a$^{+/+}$ and Tor1a$^{+/-}$ mice showed no significant difference between genotypes in frequency and amplitude (both $P>0.05$). (**F**) GABAergic mIPSCs recorded in MK-801, CNQX and TTX from SPNs of Tor1a+/+ and Tor1a+/- mice showed no significant difference between genotypes in frequency and amplitude (both P>0.05). In each group, five mice were used (N=5), and three independent electrophysiological recordings were conducted for each mouse (n=3). P<0.05 was considered to be statistically significant.

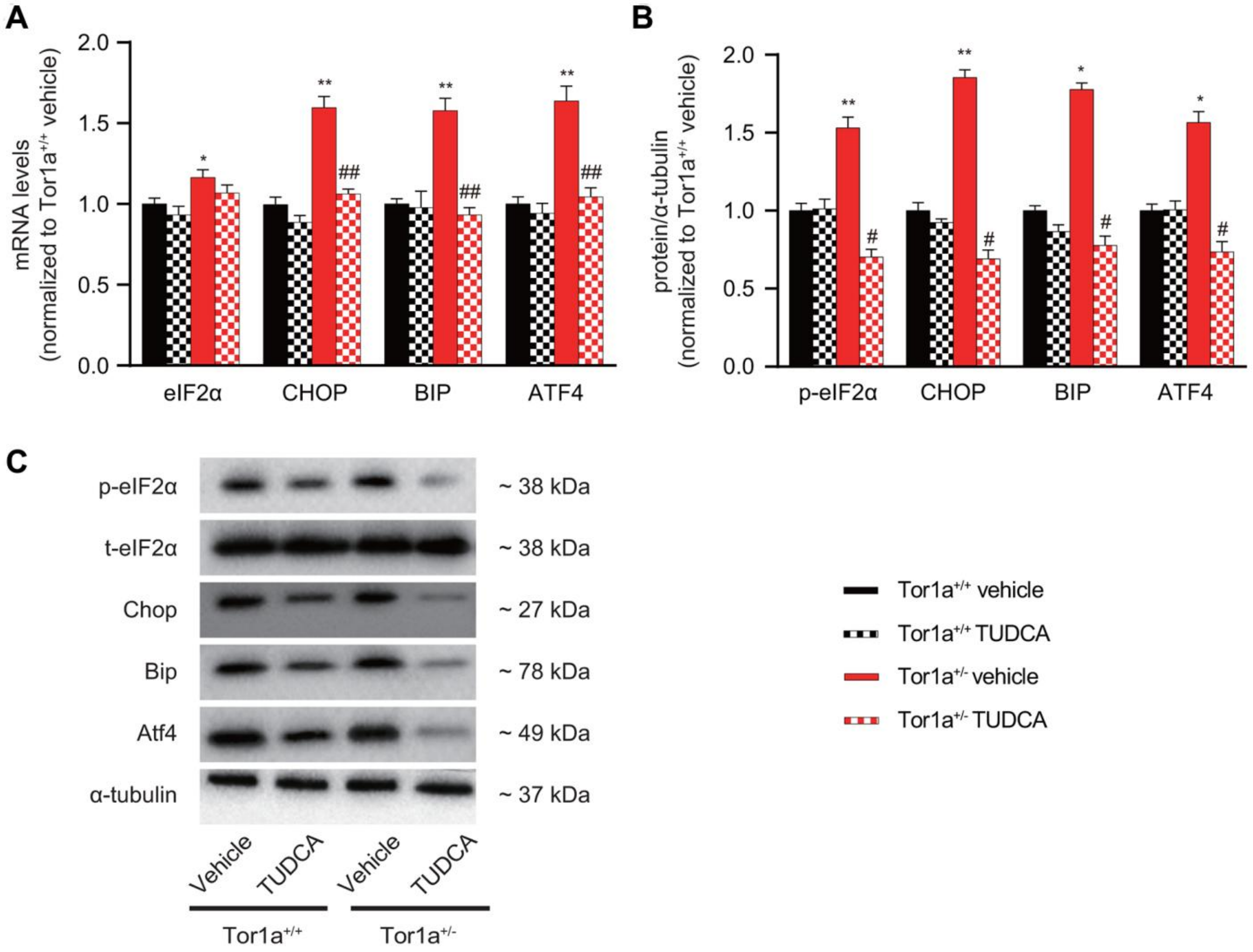

Supplementary Figure 2. TUDCA inhibits the ER stress markers in Tor1a$^{+/-}$ mice. (**A**) Levels of mRNA in striatal lysates were measured by RT-qPCR. (**B**) Quantification of protein expression in striatum ais shown (N=5 per group). Data are represented as mean ±SEM. (**C**) Representative western blots of striatal lysates. In each group, five mice were used (N=5), and three independent experiments were conducted for each mouse (n=3). *P*<0.05 was considered to be statistically significant.

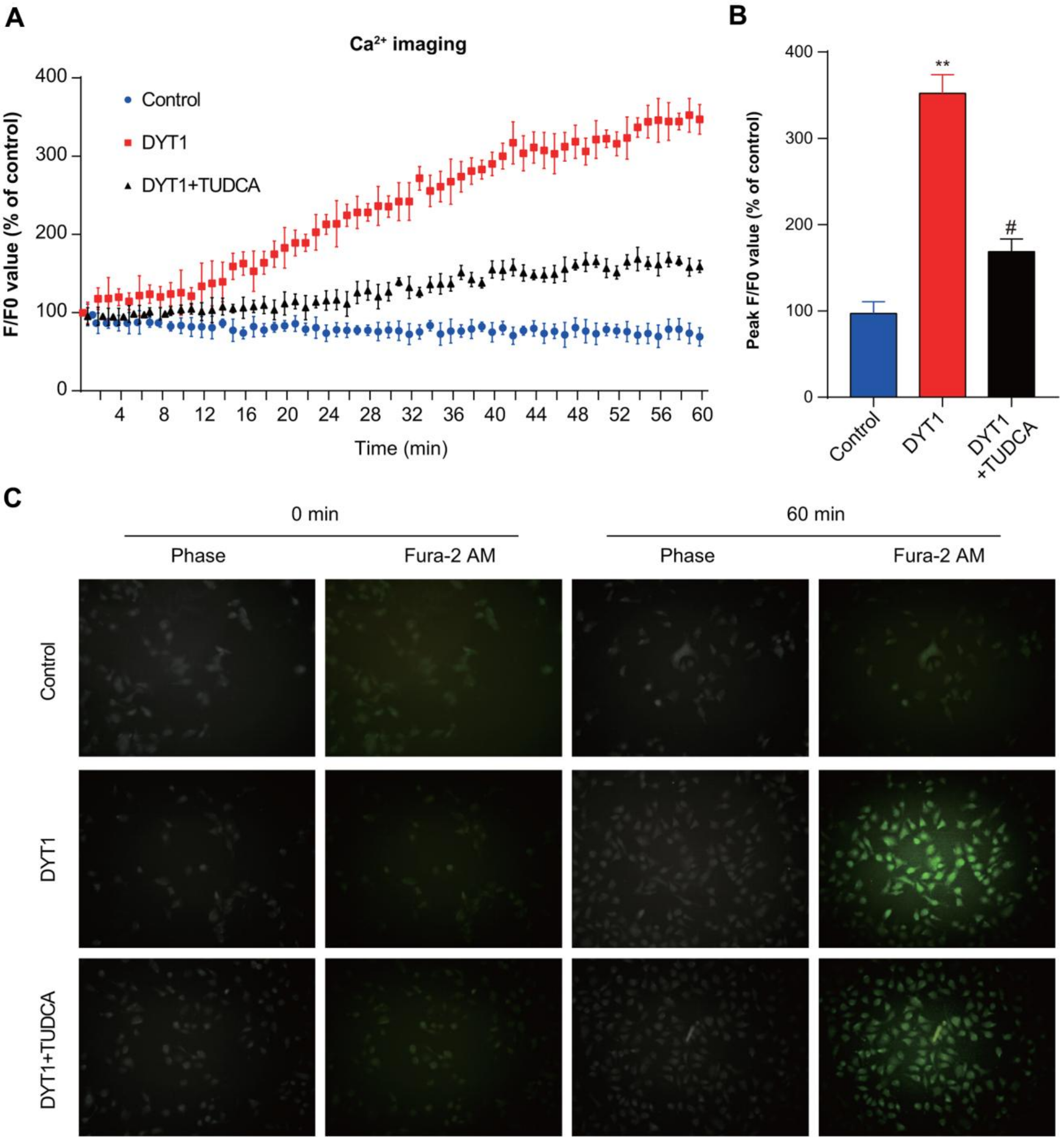

Supplementary Figure 3. TUDCA restores calcium dynamics in Tor1a$^{+/-}$ striatal spiny projection neurons (SPNs) under ER stress. (**A**) Ca^{2+} imaging in Ca^{2+}-free solution to examine the role of TUDCA *in vitro*. (**B**) Quantification of Ca^{2+} imaging shows that SPNs from Tor1a$^{+/-}$ mice revealed a significantly higher intracellular Ca^{2+} concentration than that of SPNs from Tor1a$^{+/+}$ mice. However, the intracellular Ca^{2+} release induced by Tor1a$^{+/-}$ was markedly alleviated by the ER stress inhibitor TUDCA. (**C**) Representative pictures of Ca^{2+} imaging at 0 and 60 min. In each group, five mice were used (N=5), and three independent experiments were conducted for each mouse (n=3). $P<0.05$ was considered to be statistically significant.
